# Advanced Carbon Nanomaterials for Electrochemical Sensing in the Determination of Trace V(V) Concentrations

**DOI:** 10.3390/ma19132769

**Published:** 2026-06-30

**Authors:** Malgorzata Grabarczyk, Cecylia Wardak

**Affiliations:** Department of Analytical Chemistry, Institute of Chemical Sciences, Maria Curie–Sklodowska University, 20-031 Lublin, Poland

**Keywords:** vanadium, carbon nanomaterials, electrochemical sensor, adsorptive stripping voltammetry

## Abstract

A new method is described for the determination of vanadium using adsorptive stripping voltammetry of V(V) complexed with cupferron at a CNTs/SGC electrode modified with a lead film. The CNTs/SGC electrode is based on carbon nanomaterials such as carbon nanotubes and spherical glassy carbon, which form the foundation of modern sensor technology. Optimal conditions of adsorptive voltammetric measurement were found to be modification/accumulation potential and time of −1.6 V and 60 s, respectively, and supporting electrolyte of 0.2 mol/L NaAc–HAc buffer (pH 5.3) containing 0.3 mmol/L cupferron and 0.15 mmol/L Pb(II). The response of the system was found to be linear in a range of V(V) concentrations from 0.25 nmol/L to 10 nmol/L. The detection limit was found to be 0.08 nmol/L. The selectivity of the procedure was determined by analysing the effect of other interfering ions on the vanadium analytical signal. The method was successfully validated by analysing natural environmental waters.

## 1. Introduction

The determination of metals in environmental waters is crucial for protecting human health and monitoring the state of ecosystems. The presence of metals in surface and groundwater disrupts plant growth and is toxic to fish and microorganisms. Metals enter the food chain, accumulating in the tissues of aquatic organisms, which ultimately poses a threat to consumers (including humans) as even low concentrations can cause serious illness. These metals include, among others, vanadium, which is a widely distributed element in the environment, present in soil, air and water. Its concentration in environmental waters varies and depends on geological and atmospheric factors, as well as human activity [1,2]. Generally, natural levels of vanadium in environmental waters do not pose a risk, but its concentration may increase in heavily industrialised areas. Vanadium is a key metal in industry, used mainly in the production of high-strength steel alloys (automotive, aerospace, tools) and as a catalyst (e.g., in the production of sulphuric acid). In the aquatic environment, vanadium occurs mainly in higher oxidation states, of which the pentavalent (V) form is the most soluble and mobile [3,4]. Consequently, most procedures are aimed at determining this specific form of vanadium using various methods depending on the sample matrix. The determination of vanadium(V) in environmental waters requires sensitive analytical methods due to its presence in trace quantities. The main methods include spectroscopic and chromatographic techniques, as well as electrochemical methods, primarily adsorptive stripping voltammetry (AdSV) [5,6,7]. A key parameter in stripping voltammetry is the choice of electrode material forming the basis of the working electrode, which must be a polarisable electrode and on which the analyte to be determined is concentrated. There are three main types of materials used in working electrodes: mercury, noble metals and carbon-based materials [8,9,10]. The ideal material meeting the requirements for working electrodes is mercury, which is characterised by high polarisability, i.e., the ability to accept the potential of an external source in the absence of a depolariser, which is a major advantage. This is due to the almost perfectly smooth and clean surface of mercury, which is a liquid at room temperature. Another undoubted advantage is its wide operating range in the cathodic region, i.e., on the negative potential side, which is the area where reduction processes occur (the limitation being hydrogen evolution). It is therefore not surprising that, in the case of AdSV procedures for the determination of vanadium (as with other metals), the vast majority of methods rely on mercury working electrodes. Examples of such mercury electrodes include: the hanging drop mercury electrode, the mercury film electrode, the renewable mercury film silver-based electrode, and the mercury multimode electrode. As for noble metal electrodes, these are virtually never used for vanadium determination; the literature describes only one procedure using gold microwire electrodes, which were modified with a mercury layer, so ultimately this electrode can be classified as a mercury electrode [11]. In the literature, a single procedure for the determination of V(V) can be found, using a solid bismuth microelectrode as the working electrode, as an example of a metallic electrode [12]. The third type of material currently undergoing significant development is carbon-based materials [13,14]. The most commonly used of these, due to its wide range of potentials in the cathodic and anodic regions, high chemical inertness and the ease with which its surface can be renewed by polishing, is glassy carbon (GC). Unfortunately, in the case of vanadium, as with precious metal electrodes, it is not possible to adsorb it directly onto the GC surface, which is essential for accumulation in stripping voltammetry. Therefore, GC electrodes require special modification to obtain an active surface on which V(V) adsorption occurs in the form of electrochemically active complexes. The glassy carbon was modified by depositing a film of lead or bismuth [15,16,17]. Carbon pastes are also used as carbon electrode materials; in the case of vanadium analysis, there are a few procedures that utilise them. One of these procedures involves the deposition of vanadium in the form of V(V)–alizarin red on a carbon paste electrode, whilst another involves the deposition of vanadium in the form of V(V)–alizarin violet on an acetylene black paste electrode [14,18]. Currently, carbon nanomaterials play a huge role, forming the foundation of modern sensor technology. Their unique properties ensure their widespread use across many branches of industry, including electronics, optoelectronics, battery and capacitor manufacturing, as well as in medicine for diagnostic imaging and targeted drug delivery. Thanks to their nanoscale dimensions and unique structure, they offer an extremely high surface-to-volume ratio and exceptional electronic conductivity, which has enabled carbon nanomaterials to be used in electrochemical methods as well, where they are employed in the construction of electrochemical sensors, including working electrodes used in stripping voltammetry [19,20,21]. With regard to vanadium determination, the literature describes one procedure in which a glassy carbon electrode was modified with multi-walled carbon nanotubes and the complexation occurred in the form of a V(V)–alizarin red complex [22]. To the best of our knowledge, no other AdSV procedure for the determination of V(V) utilising carbon nanomaterials for the construction of the working electrode has been described in the literature since then. In this paper, we describe a procedure for the determination of V(V) using a working electrode based on carbon nanomaterials, such as carbon nanotubes (CNTs) and spherical glassy carbon (SGC), which was modified with a lead film.

## 2. Experimental

### 2.1. Apparatus and Reagents

Suprapur or analytical grade chemicals were employed. A standard solution of 1 g L^−1^ of V(V), Pb(II) and studied foreign ions were procured from Fluka (Buchs, Switzerland). Cupferron (benzenamine, N-hydroxy-N-nitroso-ammonium salt) was obtained from Merck (Darmstadt, Germany). A solution of 1 × 10^−2^ mol L^−1^ of cupferron was prepared every day by dissolving 0.0155 g of the reagent in water in a 10 mL volumetric flask. Acetate buffers were prepared from acetic acid and sodium hydroxide (Suprapur; Merck, Darmstadt, Germany). All studies were carried out using deionized water in a laboratory purification system (Milli-Q system). Humic acid sodium salt (HA) was obtained from Merck Life Science (Poznań, Polska), and fulvic acid (FA) and natural organic matter (NOM) were obtained from the Suwannee River and purchased from the International Humic Substances Society.

All voltammetric measurement was carried out on a µAutolab (Eco Chemie, Utrecht, The Netherlands) with a personal computer operated by GPES 4.9 software. The measurements were carried out using a 10 mL quartz cell. The three-electrode array consisted of an Ag/AgCl electrode filled with saturated NaCl as a reference electrode, a platinum counter electrode, and a multi-wall carbon nanotubes/spherical glassy carbon (CNTs/SGC) electrode as a working electrode. All potentials were given relative to the Ag/AgCl electrode. The CNTs/SGC electrode was prepared using multi-walled carbon nanotubes (O.D. × I.D. × L 10 nm ± 1 nm × 4.5 nm ± 0.5 nm × 3~6 μm) purchased from Sigma-Aldrich (St. Louis, MO, USA) with ≥98% carbon basis and without functionalization. Spherical glassy carbon powder, size 0.4–12 µm, was purchased from HTW Hochtemperatur-Werkstofe GmbH (Thierhaupten, Germany). The resin (ARALDIT F, Huntsman Advanced Materials; Spring, TX, USA) was mixed with hardener (ARADUR HY 905, Huntsman Advanced Materials; Spring, TX, USA) in a 1:1 ratio.

The CNTs/SGC electrode was prepared in two stages as described in paper [23]. In the first stage, multi-walled carbon nanotubes were mixed with epoxy resin (in a mass ratio of 1:25); in the second stage, the resulting mixture was combined with spherical glassy carbon (in a mass ratio of 2:1). In the meantime, to remove air bubbles, the paste was heated to 115 °C for 10 min. and centrifuged for 10 min. at 10,000 rpm. Finally, the paste was injected under pressure into a 2 mm diameter hole previously drilled in the epoxy resin, into which a copper wire had been placed. In this form, the electrode was placed in a drying oven at 105 °C for 48 h, where it underwent its final curing. In this way, three independent electrodes were manufactured simultaneously. Before commencing measurements, the electrode was polished on sandpaper with coarse grit (P120), followed by fine-grained sandpaper (P2000). On each day of measurement, the electrode was additionally polished using 0.3 µm aluminium oxide on a Buehler polishing sponge. After each polishing stage, the electrode was rinsed in an ultrasonic bath for 30 s to remove any remaining polishing material.

**Figure 1:**
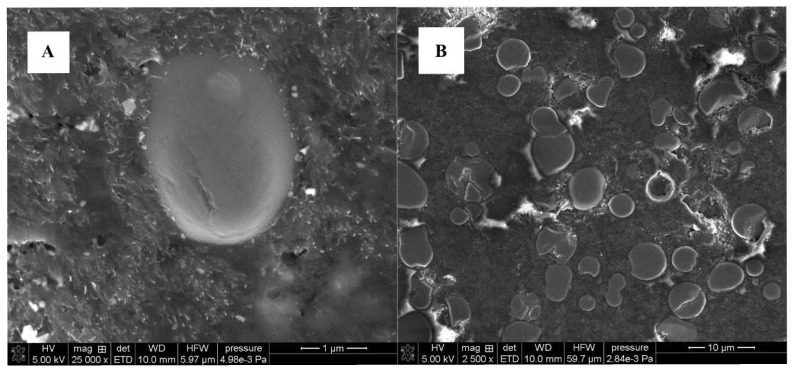
A real image of the solid CNTs/SGC electrode taken using a scanning electron microscope (Quanta 3D FEG(FEI): (**A**) magnification 25,000×; (**B**) magnification 2500×.

The morphology of the CNTs/SGC electrode was characterized using a scanning electron microscope (Quanta 3D FEG (FEI, Hillsboro, OR, USA)) and analysed at accelerating potential of 5 kV using an ETD detector in high-vacuum condition. Figure 1A (magnification 25,000×) clearly shows a particle of spherical glassy carbon situated in the centre, surrounded by numerous nanotubes embedded in resin. Figure 1B (magnification 2500×) shows numerous particles of spherical glassy carbon.

### 2.2. Analytical Procedure

Unless otherwise stated, a 0.2 mol/L NaAc–HAc buffer (pH 5.3) containing 0.3 mmol/L cupferron and 0.15 mmol/L Pb(II) was used as the supporting electrolyte for V(V) determination. The modification/accumulation step proceeded at −1.6 V for 60 s while stirring the solution, and the differential pulse stripping voltammograms after 5 s quiescence were recorded from −0.6 to −0.9 V with a scan rate of 0.0795 V/s. The modulation time and interval time were 0.002 s and 0.1 s, respectively, while the step potential and modulation amplitude were 0.00795 V and 0.1005 V, respectively. The course of the reactions occurring during the measurement procedure can be described as follows. At a potential of −1.6 V, Pb(II) is reduced to its metallic form; in accordance with the mechanism of V(V) reduction in solutions described in [24], it accumulates in its reduced form at the same time. Subsequently, when the potential changes to −0.6 V, vanadium reverts to its oxidised form, forming a complex with cupferron in the near electrode layer. As a result of the potential change during voltammetric recording in the range from −0.6 to −0.9 V, this complex is reduced in accordance with the mechanism described by J. Wang and colleagues [25].

The peak currents observed on the voltammograms within the designated concentration range was proportional to the concentration of V(V) ions in the solution.

After each measurement, the CNTs/SGC electrode was electrochemically cleaned by applying two successive potentials: first −1.6 V for 5 s, followed by 0.2 V for 5 s. During the first one, any residues from the measurement are reduced, and then, as a result of the potential changing to positive, they are oxidised and thus removed from the electrode surface into the solution. After this cleaning, the measurement cell was rinsed with distilled water and then a new solution was introduced, from which the next measurement was carried out. All measurements were executed at room temperature in the presence of oxygen. When using a lead film, there is no need to remove oxygen from the solution, which is a major advantage. This is because the reduction of oxygen on lead occurs in a single step within a potential range of 0 to –0.2 V, meaning that oxygen does not interfere with measurements carried out using a lead film.

Every day, before measurement, the electrode was polished with a 0.3 µm suspension of aluminium oxide on a Buehler polishing pad for 30 s and then immersed in an ultrasonic bath for 30 s to remove aluminium oxide.

## 3. Results and Discussion

### 3.1. Modification of the CNTs/SGC Electrode

No vanadium signal was observed when attempting to accumulation it in the form of a complex with cupferron directly on the CNTs/SGC electrode, which confirmed earlier reports in the literature in which glassy carbon electrodes were modified with a lead or bismuth film to ensure effective concentration of V(V) complexes on their surface. Therefore, the initial aim of our research was to select a method for modifying the CNTs/SGC electrode. One of the simplest yet most effective modification methods is the deposition of a metallic film, most commonly bismuth or lead, onto the electrode surface. Methods for depositing films (modifying layers) onto electrodes in voltammetry are divided into in situ (at the measurement site) and ex situ (off-site) methods. In situ film deposition (at the measurement site) involves depositing a modifying layer directly in the electrochemical measurement cell at the same time as the voltammetric analysis; metal cations are reduced and deposited as a metallic film (e.g., Pb, Bi) on the electrode during the initial concentration stage. In this case, the film is removed after each measurement and a new film is deposited for each measurement, which allows for renewable and repeatable electrode surfaces, ensuring good measurement precision. Ex situ film deposition (outside the measurement site) involves first generating the film by reducing metal cations outside the measurement vessel and then transferring the electrode to the base electrolyte to perform the measurement. This is a more time-consuming method, which increases the total measurement time and is less recommended. We therefore chose, in our research, to modify the CNTs/SGC electrode by applying a metallic film using the in situ method. We used a lead film as the metallic film.

The optimisation of the parameters for modifying the CNTs/SGC electrode with a lead film was carried out for a solution with a constant V(V) concentration of 5 nmol/L and a constant cupferron concentration of 0.3 mmol/L, and Pb(II) concentration of 0.15 mmol/L.

*Base electrolyte*: Based on data from the literature, acetate buffers with varying pH values ranging from 3 to 6 were selected for testing. For each of these, a measurement was carried out and the vanadium peak current was determined. Analysing the relationship between the maximum peak current and the pH value, it was found that as the pH increased, the peak rose to a value of 5.3 and then remained constant. Consequently, a buffer with a pH of 5.3 was used as standard for further investigations. Its concentration in the measuring cell was 0.2 mol/L.

**Figure 2:**
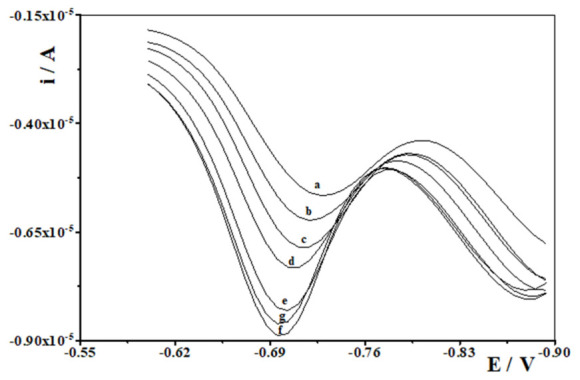
Voltammetric curves recorded at various modification/accumulation potentials: −1.1 V (a), −1.2 V (b), −1.3 V (c), −1.4 V (d), −1.5 V (e), −1.6 V (f), −1.7 V (g). A 0.2 mol/L NaAc–HAc buffer (pH 5.3) containing 0.3 mmol/L cupferron and 0.15 mmol/L Pb(II) was used as the supporting electrolyte for the determination of 5 nmol/L V(V).

*Lead film formation (modification) potential and V(V) accumulation potential*: In most voltammetric procedures, the stages of film formation and accumulation of the analyte on the film take place at two different potentials; however, there are also procedures in which both processes occur simultaneously at a single potential. Following preliminary measurements, it was found that in the system we propose, it is possible to use a single common potential for both processes. The effect of the modification/accumulation potential on the vanadium signal was investigated in the range from −1.1 V to −1.7 V, varying its value by 0.1 V. It was observed that as the negative potential value increased to −1.6 V, the peak grew and the peak potential shifted towards less negative potential values, as shown in Figure 2. At a potential of −1.7 V, the peak potential no longer shifted and its value began to decrease. A lead film formation potential of −1.6 V, with simultaneous V(V) accumulation, was selected as optimal. The next optimisation step was to investigate the effect of the duration of the selected modification/accumulation potential on the magnitude of the vanadium peak current. It was observed that the peak increased with increasing time up to 60 s.

**Figure 3:**
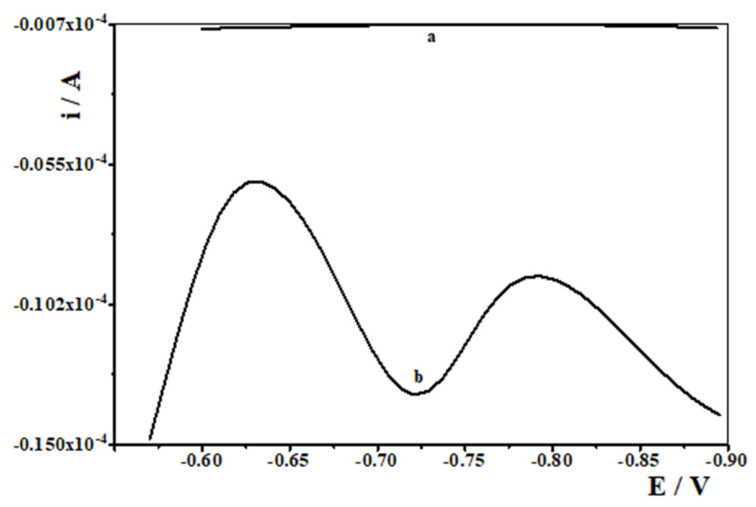
Voltammograms recorded from solutions of: (a) 0.2 mol/L NaAc–HAc buffer (pH 5.3) + 0.3 mmol/L cupferron + 2.5 nmol/L V(V); (b) as (a) +0.15 mmol/L Pb(II). Modification/accumulation potential: −1.6 V for 60 s.

**Figure 4:**
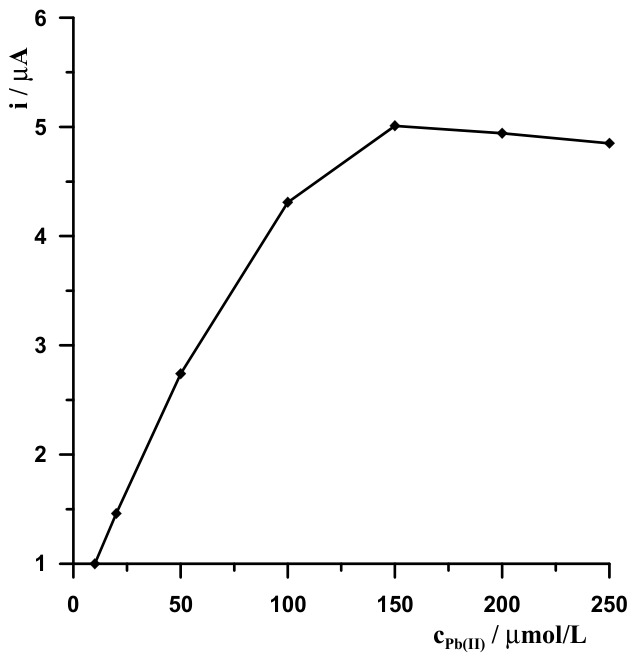
The effect of Pb(II) concentration on the peak current response of 5 nmol/L V(V). Modification/accumulation potential: −1.6 V for 60 s. A 0.2 mol/L NaAc–HAc buffer (pH 5.3) containing 0.3 mmol/L cupferron was used as the supporting electrolyte.

*Pb(II) concentration in solution*: Another important parameter to be taken into account when modifying an electrode with a metallic film is the concentration of metal ions in the solution from which the film is generated. Figure 3 shows the voltammetric signal obtained for V(V) for a lead-film-modified (b) and an unmodified CNTs/SGC electrode (a). As can be seen, when the CNTs/SGC electrode is not modified with lead film, no vanadium signal is observed and the recorded currents are slight. In our procedure, we investigated the effect of Pb(II) ion concentration on the vanadium peak current. The experiments were carried out using a solution in which the Pb(II) concentration varied between 10 and 250 µmol/L. The vanadium signal only began to appear at a Pb(II) concentration of 20 µmol/L in the solution and increased as the concentration rose to 150 µmol/L (selected as the most optimal), and then decreased slightly at higher concentrations (Figure 4).

### 3.2. Cupferron as a Complexing Agent for V(V)

**Figure 5:**
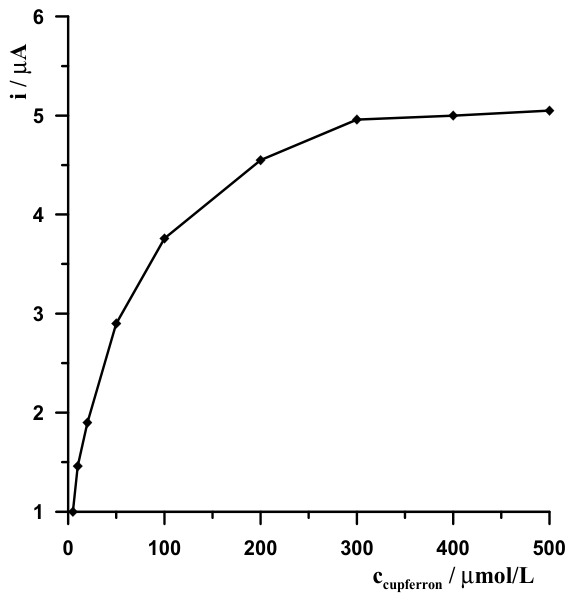
The effect of cupferron concentration on the peak current response of 5 nmol/L V(V). Modification/accumulation potential: −1.6 V for 60 s. A 0.2 mol/L NaAc–HAc buffer (pH 5.3) containing 0.15 mmol/L Pb(II) was used as the supporting electrolyte.

The most suitable medium for the voltammetric determination of V(V) in the adsorption–accumulation variant is an organic complexing medium. Numerous complexing agents tested for this purpose can be found in the literature, such as catechol [26], chromoxane cyanine R [27], pyrocatechol [28], alizarin violet [18], gallic acid [11], quertic–5–sulfonic [29], pyrogallol [30], 2,3-dihydroxybenzaldehyde [31] or 2,3–dihydroxynaphthalene [32]. However, by far the best results were obtained using cupferron and chloranilic acid, as reflected in the largest number of V(V) determination procedures using precisely these complexing agents [15,16,17,33,34,35,36,37,38]. Of these two complexing agents, cupferron enabled lower detection limits to be achieved; furthermore, it is widely available and exhibits high selectivity, forming stable, electrochemically active complexes with selected metal ions. It acts as a bidentate ligand, binding to the metal cation via two oxygen atoms and forming a stable, five-membered chelate ring. Therefore, in our procedure using a lead-film-modified CNTs/SGC electrode, we opted to use cupferron as the complexing agent for V(V). The effect of cupferron concentration on the V(V) analytical signal is shown in Figure 5; following analysis of the results obtained, a concentration of 0.3 mmol/L is recommended as optimal.

### 3.3. Calibration and Detection Limit

**Figure 6:**
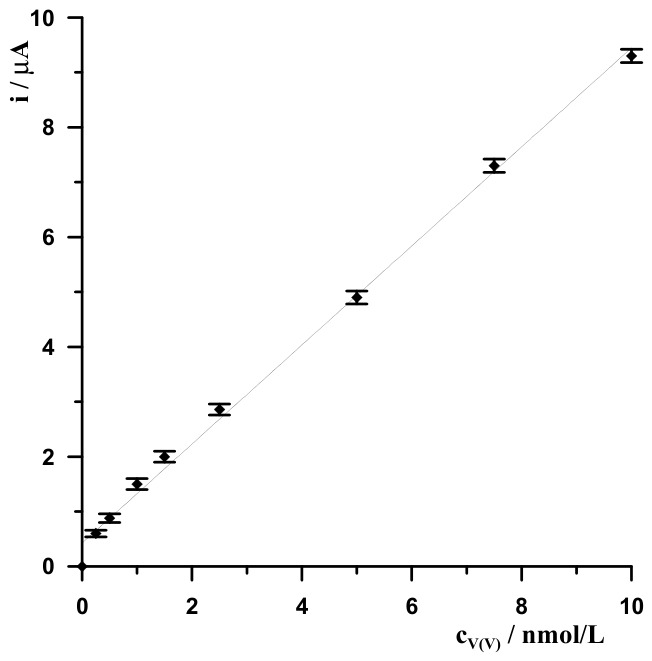
The calibration graph performed using a lead-film-modified CNTs/SGC electrode for solution: 0.2 mol/L NaAc–HAc buffer (pH 5.3) + 0.3 mmol/L cupferron + 0.15 mmol/L Pb(II) + V(V) in the concentration range of 0.25–10 nmol/L.

**Figure 7:**
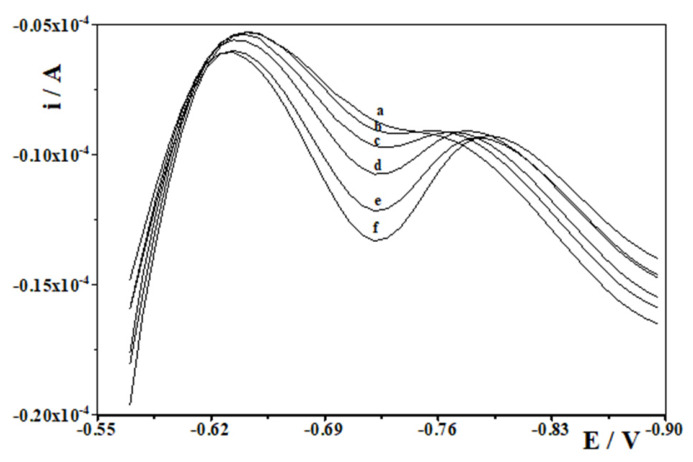
A series of voltammograms obtained using a lead film-modified CNTs/SGC electrode for low V(V) concentrations. (a) Blank and (b) 0.25, (c) 0.5, (d) 1.0, (e) 1.5, (f) 2.5 nmol/L V(V). Modification/accumulation potential: −1.6 V for 60 s. A 0.2 mol/L NaAc–HAc buffer (pH 5.3) containing 0.15 mmol/L Pb(II) and 0.3 mmol/L cupferron was used as the supporting electrolyte.

**Figure 8:**
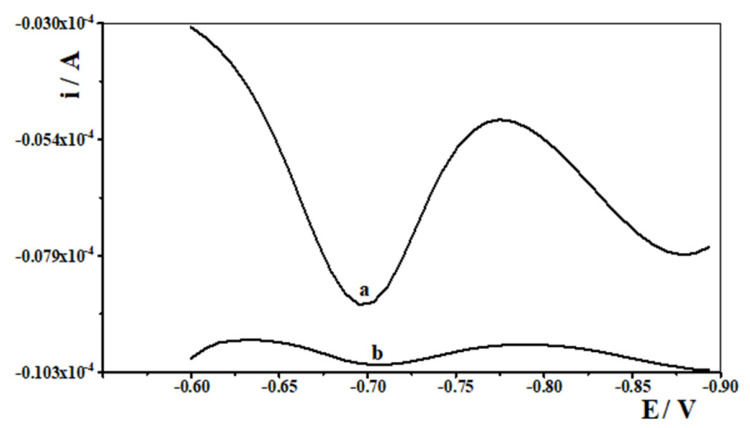
A voltammograms obtained using a lead film-modified CNTs/SGC electrode (a) and GC electrode (b) for 2.5 nmol/L V(V) concentrations. Modification/accumulation potential: −1.6 V for 60 s. A 0.2 mol/L NaAc–HAc buffer (pH 5.3) containing 0.15 mmol/L Pb(II) and 0.3 mmol/L cupferron was used as the supporting electrolyte.

Once the optimum parameters for the analysis were chosen, the linear range for the V(V)–cupferron complex was specified. Calibration was performed using a lead-film-modified CNTs/SGC electrode with a previously optimised solution composition: 0.2 mol/L NaAc–HAc buffer (pH 5.3) containing 0.3 mmol/L cupferron and 0.15 mmol/L Pb(II), to which increasing concentrations of V(V) were subsequently added. The voltammetric measurement was carried out using the stripping voltammetry technique based on the following protocol: modification/accumulation step at –1.6 V for 60 s, followed by a 5-s equilibration time, and recording of the voltammogram resulting from the potential shift in the cathodic direction. The calibration graph was linear from 0.25 nmol/L do 10 nmol/L and obeyed the equation y = 0.899x + 0.388, where y is the peak current (µA) and x is the V(V) concentration (nmol/L); the correlation coefficient (r) was 0.996. Figure 6 shows the calibration curve with error bars marked; the slope and intercept were 0.899 and 0.388, respectively. The voltammograms recorded in the absence of V(V) and in the presence of low concentrations of V(V) in the range from 0.25 nmol/L to 2.5 nmol/L are shown in Figure 7. The relative standard deviation for a V(V) concentration of 2.5 nmol/L was 3.3% (n = 5). The LOD estimated from 3 times the standard deviation for the lowest studied V(V) concentration equal to 0.25 nmol/L was about 0.08 nmol/L and the LOQ estimated from 10 times the standard deviation was 0.26 nmol/L Figure 8 shows the voltammograms recorded under identical measurement conditions for the CNTs/SGC and GC electrodes. As can be seen, the signal recorded using the CNTs/SGC electrode (voltammogram a) is eight times higher than that for the GC electrode (voltammogram b). This clearly confirms that a mixture of carbon nanomaterials, such as CNTs and SGC, allows for an increase in the sensitivity of the determinations and, consequently, a reduction in the limit of detection. This can be explained by the fact that the spherical glassy carbon particles visible in the image (Figure 1B) act as microelectrodes, ensuring high sensitivity through rapid mass transport. Spherical diffusion occurs in their vicinity, which delivers the analyte much faster than the linear diffusion occurring on conventional electrodes, such as glassy carbon. Additionally, the presence of carbon nanotubes, which are responsible for faster electron transfer in electrochemical sensors, improves the signal to background ratio.

### 3.4. The Repeatability and Precision

**Figure 9:**
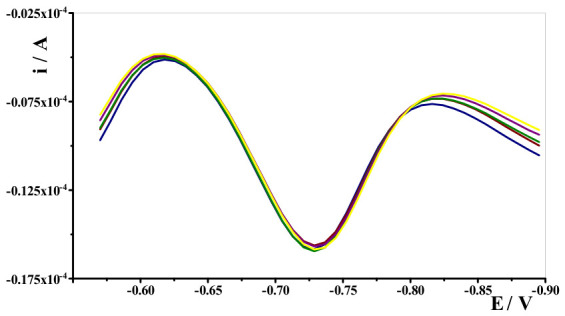
Voltammetric curves recorded over five consecutive days: blue (day 1), red (day 2), green (day 3), magenta (day 4) and yellow (day 5). Modification/accumulation potential: −1.6 V for 60 s. A 0.2 mol/L NaAc–HAc buffer (pH 5.3) containing 0.3 mmol/L cupferron and 0.15 mmol/L Pb(II) was used as the supporting electrolyte for the determination of 5 nmol/L V(V).

The short-term repeatability and between-day precision of the analytical signal for V(V) on a lead-film-modified CNTs/SGC electrode was examined. The short-term repeatability was assessed for five different samples using a supporting electrolyte containing 5 nmol/L of V(V) on a single day, The between-day precision was tested by examining the reproducibility of the peak height for 5 nmol/L V(V). Five identical measurements were made day after day, and the RSD was calculated based on the obtained peaks, which was 3.8%; the recorded voltammograms are shown in Figure 9. Fresh solutions and freshly cleaned electrode surfaces were used every day.

Electrode-to-electrode variability was also assessed by performing three identical measurements for each of three independently fabricated CNTs/SGC electrodes. For each freshly polished electrode, fresh, identical 5 nmol/L V(V) solutions were used, and an RSD of 4.8% was obtained.

### 3.5. Selectivity

The effect of various ions on V(V) determination was investigated by comparing the signal intensity of 5 nmol/L V(V) recorded in the absence of foreign ions and in their presence at 50-, 100-, 200-fold excesses, whilst maintaining identical measurement conditions. The interference threshold was set at a level above ±5% of the vanadium peak current change. It was established that the presence of As(V), Bi(III), Ca(II), Cd(II), Co(II), Cr(III), Cr(VI), Cu(II), Fe(III), K(I), Mg(II), Mn(II), Na(I), Ni(II) Ti(IV), W(VI) and Zn(II) in a 200-fold excess does not interfere with the vanadium signal. The most interfering ions were Al(III), Mo(VI) and U(VI). In the case of a 50-fold excess of Al(III), a 20% reduction in the vanadium signal was observed, and in the case of a 100-fold excess, the signal was further reduced to 50%. In the case of a 100-fold excess of Mo(VI), a 50% increase in the signal is observed, and in the case of a 200-fold excess, the signal increased by 100%. In the case of a 100-fold excess of U(VI), a 50% reduction in the vanadium signal was observed, and in the case of a 200-fold excess, the signal was further reduced to 30%. The influence of chloride, sulphate, bicarbonate, carbonate, nitrate, and phosphate on the analytical V(V) peak was also examined, and it was found that their presence in the solution, even at a concentration of 0.01 mol/L, did not interfere with the voltammetric signal.

**Figure 10:**
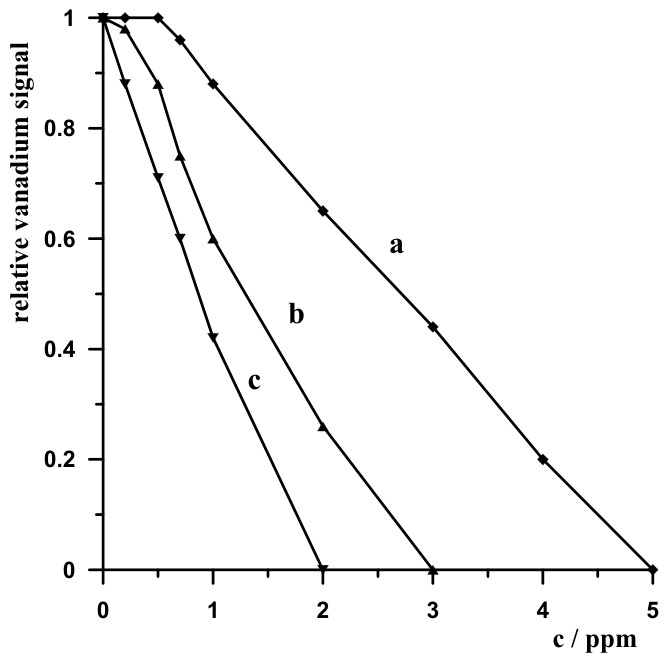
The influence of HA (a), FA (b) and NOM (c) on the relative signal of 5 nmol/L V(V). Modification/accumulation potential: −1.6 V for 60 s. A 0.2 mol/L NaAc–HAc buffer (pH 5.3) containing 0.3 mmol/L cupferron and 0.15 mmol/L Pb(II) was used as the supporting electrolyte.

The effect of organic substances potentially present in environmental waters, such as natural organic matter (NOM) or humic acids (HA) and fulvic acids (FA), is shown in Figure 10. As can be seen, NOM had the greatest interfering effect, followed to a lesser extent by FA, whilst HA had the lowest effect on the voltammetric signal V(V); signal disappearance occurred in their presence of only 3 ppm, 4 ppm and 5 ppm, respectively. This is an advantage of the proposed procedure because, in voltammetric procedures for the determination of V(V) in which the effect of these substances was investigated, a complete disappearance of the signal was observed in the presence of concentrations as low as 0.5–1 ppm.

### 3.6. Single-Level Spike-Recovery Study

The proposed procedure using a lead-coated CNTs/SGC electrode was evaluated by carrying out V(V) determinations on real environmental water samples before and after spiking with V(V) at a concentration of 2.5 nmol/L. The real water samples were analysed one hour after the addition of vanadium ions and a two-fold dilution. The deposition time and potential, as well as the voltammetric parameters, were the same as those used in the standard method. The concentration of V(V) was determined using the single-level spike-recovery study, and Table 1 shows the precision and recovery data obtained for the environmental water samples. Acceptable recovery was obtained for selected diluted water samples spiked at one concentration.

## 4. Conclusions

The research presented in this article focuses on the determination of trace concentrations of V(V) using the stripping voltammetry method with cupferron as a complexing agent. The working electrode used was an electrode developed in our laboratory based on carbon nanomaterials, namely carbon nanotubes and spherical glassy carbon (CNTs/SGC), which was modified with a lead film. This electrode was previously used successfully to determine trace amounts of various metal ions. The novelty of the present study was its first-time use as a substrate for forming a lead film simultaneously with the accumulation of the analyte of interest, which was vanadium. In the vast majority of voltammetric procedures, V(V) is deposited as a result of its adsorption in the form of a V(V)-complexing agent, whereas in the procedure we propose, V(V) is deposited in metallic form and then, following a change in potential, converts to its ionic form, forming an electrochemically active complex with cupferron. Based on the results of the experiments presented in the manuscript, the following key summary and conclusions can be drawn:

- A lead-film-modified CNTs/SGC electrode may offer a valuable alternative for the determination of V(V) using stripping voltammetry;
- A low detection limit of 0.08 nmol/L was achieved, combined with good repeatability and precision;
- The procedure exhibits good selectivity for the determination of V(V) relative to many other metal ions;
- The developed procedure is simple and rapid; thanks to the combination of electrode modification and analyte accumulation in a single step, the total measurement time is just over a minute;
- As demonstrated, direct analysis of environmental water samples is possible.

## Figures and Tables

**Table 1:** Single-level spike-recovery study for V(V) obtained on natural water samples.

Sample	Concentration of V(V) [nmol/L]			Recovery [%]	RSD (n = 3) [%]
	Original	Added	Found		
River water	-	2.50	2.42	96.8	5.2
Lake water	-	2.50	2.38	95.2	6.1
Mineral water	-	2.50	2.47	98.8	4.4

## Data Availability

The original contributions presented in this study are included in the article. Further inquiries can be directed to the corresponding author.
